# Maternal Stressful Life Events during Pregnancy and Atopic Dermatitis in Children Aged Approximately 4–6 Years

**DOI:** 10.3390/ijerph18189696

**Published:** 2021-09-15

**Authors:** Camilla C. Senter, Nicole R. Bush, Christine T. Loftus, Adam A. Szpiro, Annette L. Fitzpatrick, Kecia N. Carroll, Kaja Z. LeWinn, W. Alex Mason, Sheela Sathyanarayana, Oluwatobiloba A. Akingbade, Catherine J. Karr

**Affiliations:** 1 Department of Epidemiology, University of Washington, Seattle, WA 98195, USA; fitzpal@uw.edu (A.L.F.); ckarr@uw.edu (C.J.K.); 2 Department of Psychiatry and Behavioral Sciences, University of California, San Francisco, CA 94143, USA; Nicole.Bush@ucsf.edu (N.R.B.); Kaja.LeWinn@ucsf.edu (K.Z.L.); 3 Department of Pediatrics, University of California, San Francisco, CA 94143, USA; 4 Department of Environmental and Occupational Health Sciences, University of Washington, Seattle, WA 98195, USA; cloftus@uw.edu (C.T.L.); sheela.sathyanarayana@seattlechildrens.org (S.S.); oaaking@uw.edu (O.A.A.); 5 Department of Biostatistics, University of Washington, Seattle, WA 98195, USA; aszpiro@uw.edu; 6 Department of Family Medicine, University of Washington, Seattle, WA 98195, USA; 7 Department of Global Health, University of Washington, Seattle, WA 98195, USA; 8 Department of Pediatrics, Vanderbilt University Medical Center, Nashville, TN 37232, USA; kecia.carroll@mssm.edu; 9 Department of Preventive Medicine, University of Tennessee Health Science Center, Memphis, TN 38163, USA; wamason@uthsc.edu; 10 Center for Child Health, Behavior, and Development, Seattle Children’s Research Institute, Seattle, WA 98101, USA; 11 Department of Pediatrics, University of Washington, Seattle, WA 98195, USA

**Keywords:** prenatal stress, stressful life events, atopic dermatitis, child sex, history of atopy, resilience

## Abstract

The prevalence of atopic dermatitis (AD) in children has steadily increased over time, yet it remains largely unknown how maternal factors during pregnancy are associated with child AD. Few studies have specifically assessed the relationship between prenatal stress and child AD, with inconsistent findings. In this prospective cohort study following 426 mother-child dyads from pregnancy to middle childhood, women reported stressful life events (SLEs) experienced during the 12 months before delivery and AD outcomes in children aged approximately 4–6 years, including current, location-specific, and ever AD. We used Poisson regression to estimate risk ratios (RRs) and corresponding 95% confidence intervals (CIs) associated with a 1-unit increase in prenatal SLEs, adjusting for potential confounders. We also assessed whether the association between prenatal SLEs and child AD was modified by child sex, history of maternal atopy, or prenatal maternal resilient coping. The mean (standard deviation) of prenatal SLEs reported in the overall sample was 1.4 (1.6), with 37.1% of women reporting none. A 1-unit increase in prenatal SLEs was not significantly associated with current AD (RR: 1.08, 95% CI: 0.89, 1.31), location-specific AD (RR: 1.09, 95% CI: 0.78, 1.52), or ever AD (RR: 0.97, 95% CI: 0.87, 1.09). We did not find evidence of effect modification. Findings from this study suggest no association between prenatal SLEs and AD in middle childhood, although larger longitudinal studies with enhanced case definition and higher variability of SLE experience may more fully inform this question.

## 1. Introduction

Atopic dermatitis (AD) is a chronic inflammatory skin condition characterized by itchy rash, red blistering lesions, and sensitive dry skin. It affects approximately 15–20% of children worldwide [1] and can negatively impact quality of life through decreased self-esteem, lower work or school productivity, and loss of sleep. In 2015, AD was associated with almost USD 5.3 billion in health care costs annually in the U.S., placing a significant economic burden on patients and their families [2]. It has previously been suggested that modifiable maternal factors during pregnancy may influence child AD development, including prenatal smoking [3], omega-6 polyunsaturated fatty acid status [4], and maternal stress [5,6,7]. Prenatal maternal stress may play a particularly significant role through increased maternal cortisol production [8,9,10], which modulates fetal immune system development within this crucial offspring developmental window.

Few studies have assessed the relationship between prenatal stress and child AD, the majority of which have been conducted in Europe, and findings are not consistent. Positive associations have been reported when defining prenatal stress as high job strain [11], bereavement [5], depression or anxiety [9,12,13], and, most strongly, stressful life events (SLEs) during pregnancy [6,7]. One study found that children whose mothers experienced one or more SLEs during pregnancy versus none had a 53% higher odds (95% confidence interval: 1.11, 2.10) of developing AD [6]. Another analysis reported an odds ratio of 4.19 (95% confidence interval: 1.97, 8.89) comparing children of mothers experiencing three or more prenatal SLEs to children of mothers experiencing no prenatal SLEs [7]. Findings on the relationship between prenatal stress and child AD have been inconsistent, however, as one study found no evidence of an association between prenatal anxiety, a measure of psychological functioning, and child AD [14], and a recent analysis similarly suggested a null relationship between prenatal psychological distress and child AD [15]. In addition to there being little existing work on maternal exposures to SLEs during pregnancy, the literature is also limited in that few previous studies have assessed the relationship between prenatal stress and AD in children older than 4 years [6,7,16]. AD remission is common and can occur at a young age, and AD persisting into middle childhood may be linked to more severe disease [17].

In addition, the relationship between maternal prenatal SLEs and child AD may differ when stratified across levels of potential effect modifiers, but this has rarely been explored in the literature. First, child sex may modify the relationship, as female children have been hypothesized to have increased vulnerability to developmental programming and changes in maternal stress-sensitive cortisol levels compared to male children [18,19]. Second, two previous studies have reported a modifying effect of maternal history of atopy (defined as ever AD, asthma, or allergic rhinitis), specifically on the associations between prenatal stress and asthma [7], and postpartum depression and child AD [20]. Finally, greater resilient coping in response to prenatal stressful experiences may attenuate the relationship between prenatal SLEs and child AD, as previous work has reported the buffering effects of maternal resilience on a range of offspring outcomes [21,22], although not yet on AD specifically.

This multicenter prospective cohort study is the first to examine the association between prenatal stress and child AD in the U.S and the first to explore potential buffering by maternal resilience. Specifically, the primary aim of this study was to assess the relationship between maternal exposures to SLEs during pregnancy and children’s current AD, location-specific AD, and ever AD in children aged approximately 4–6 years. As a secondary aim, we examined whether the association between prenatal SLEs and child AD was modified by child sex, maternal history of atopy, or prenatal maternal resilient coping.

## 2. Materials and Methods

### 2.1. Data Source and Study Population

The participants of this study originally participated in the Global Alliance to Prevent Prematurity and Stillbirth (GAPPS) study, a prospective prenatal cohort that collects demographic information, health history, and biospecimens from pregnant women aged 18 years or older at each trimester visit and delivery. Women who participated prenatally in the GAPPS study in Washington state between 2011 and 2016 and consented to have their information and specimens stored in a repository were later invited to join the Environmental influences on Child Health Outcomes (ECHO) PATHWAYS study. ECHO PATHWAYS is a consortium of three prenatal cohorts, including GAPPS, and aims to determine prenatal factors that affect child health outcomes, including airway health and neurodevelopment. This study was reviewed and approved by the University of Washington Human Subjects Division Institutional Review Board. Informed consent was obtained at initial GAPPS enrollment and at the ECHO PATHWAYS first study visit (child aged 4 to 6-year visit).

The sample of the current study consisted of mother-child dyads who were enrolled prenatally in GAPPS at three different sites in Washington (University of Washington Medical Center, Seattle; Swedish Medical Center, Seattle; Yakima Valley Memorial Hospital, Yakima) and were later recruited for ECHO PATHWAYS postnatal follow-up. Participants were included in analysis if they completed questionnaires assessing prenatal SLEs and child AD during the ECHO PATHWAYS clinic visit targeting children approximately aged 4 to 6-years. Children younger than 4 years were excluded from our sample for two reasons. First, we were primarily interested in AD persisting into middle childhood, which may be indicative of more severe disease. Second, the survey instruments used for exposure and outcome assessment were administered when children were approximately 4–6 years old. After excluding children who were younger than 4 years or older than 7.5 years at the time of the child follow-up visit, the available study sample was 433 mother–child dyads.

### 2.2. Assessment of Exposure

During the age 4 to 6-year child visit, women completed the self-administered 14-item Centers for Disease Control and Prevention (CDC) Pregnancy Risk Assessment Monitoring System (PRAMS) SLE survey [23]. Implemented in the United States in 1987, PRAMS is a risk factor surveillance system that collects information on maternal behaviors and experiences before and during pregnancy as well as during early childhood. The reliability and validity of self-reported PRAMS survey indicators has previously shown to be high [24]. The SLE questionnaire contains 14 questions regarding the mother’s experience of a set of stressful events in the 12 months prior to delivery (yes/no). These include events in domains including relationship problems, housing or financial issues, legal problems, and illness or death of a loved one. The PRAMS survey was originally designed to assess SLEs in the year prior to delivery in order to compare differences by baseline pre-pregnancy alcohol and tobacco use [23]. An exposure variable was calculated by adding the number of SLE questions answered affirmatively to create a composite sum score (possible range, 0–14), an operationalization that has been used previously and reflects the current lack of a standard accepted approach to categorizing SLE responses [25].

### 2.3. Assessment of Outcomes

All AD outcomes were defined using the International Study of Asthma and Allergies in Childhood (ISAAC) survey [26], which was completed by the child’s mother during the approximately aged 4 to 6-year visit. ISAAC, which was formed in 1990 from the merging of two multinational asthma and allergies projects in New Zealand and Germany, is an initiative that aims to assess prevalence, severity, and etiological trends of asthma and allergic diseases. ISAAC survey domains include asthma, wheezing, rhinitis, and eczema. The development of the ISAAC survey was informed by dermatologists and pediatricians and is a validated assessment of AD in children [26]. AD outcomes were characterized using previously described definitions [4]. Current AD was defined as positive responses to ISAAC questionnaire items, “has your child ever had an itchy rash which was coming and going for at least 6 months?” and “has your child had this itchy rash at any time in the past 12 months?” Location-specific AD, considered a more specific characterization than current AD, was defined as having current AD and additionally answering affirmatively to the question, “has this itchy rash at any time affected any of the following places: the folds of the elbows, behind the knees, in front of the ankles, under the buttocks, or around the neck, ears or eyes?” Finally, we considered a secondary outcome of ever AD to be a positive response to the question, “has your child ever had eczema?”

### 2.4. Covariates

Data were collected on sociodemographic information, health history, and pregnancy characteristics from both prenatal and follow-up visits. We determined potential confounding and precision variables to include in multivariable models a priori. The child covariates included were sex (male, female) and age at follow-up visit (years). The following maternal covariates were included: history of atopy (self-reported of ever AD, asthma, or allergic rhinitis; yes, no), age at delivery (years), current stress (maternal score on Cohen’s Perceived Stress Scale at the approximately aged 4 to 6-year visit; possible range, 0–16), prenatal completed level of education (less than high school degree, high school graduate or GED, some college or technical/vocational school graduate, college degree or more), race (White, Black or African American, Asian, American Indian/Alaskan Native, multiple race, other), ethnicity (Hispanic/Latino, not Hispanic/Latino), prenatal smoking (yes, no), prenatal farm animal exposure (yes: ≥1 time per week, no: <1 time per week), prenatal cat or dog ownership (yes, no), delivery type (vaginal, caesarian section), breastfeeding duration (did not breastfeed, >0 to <6 months, ≥6 months), and prenatal antibiotics use (yes, no). In assessing whether the association between prenatal SLEs and child AD differed by prenatal resilient coping, we defined coping categories according to prenatal responses on the validated Brief Resilient Coping Scale (BRCS). As previously described, low, medium, and high resilient coping were categorized as BRCS scores of 4–13, 14–16, and 17–20, respectively [27]. Other covariates included history of paternal atopy (maternal report of ever AD, asthma, or allergic rhinitis in biological father; yes, no), prenatal household income (<USD 50,000, ≥USD 50,000 to <USD 80,000, ≥USD 80,000), recruitment site (Seattle, Yakima), and other children present in the home at time of follow-up visit (yes, no).

### 2.5. Statistical Analysis

We summarized maternal and child characteristics by reporting medians and interquartile ranges (IQRs) for continuous covariates and frequencies and sample percentages for categorical covariates. Distributions were described in the overall sample as well as in groups defined by the median prenatal SLEs experienced. We also reported frequencies for each type of individual SLE experienced prenatally across the cohort.

We used multivariable Poisson regression with robust standard errors to estimate risk ratios (RRs) and corresponding 95% confidence intervals (CIs). In primary analyses, we measured associations between each 1-unit increase in prenatal SLEs and presence of primary outcomes (current AD, location-specific AD) using a staged model approach. The minimal model was adjusted for child sex, child age, maternal education, household income, maternal race, maternal ethnicity, and maternal history of atopy. The main model included all covariates considered relatively strong confounders or precision variables. This model additionally controlled for maternal age at delivery, maternal current stress, recruitment site, other children in home, and prenatal farm animal exposure. The extended model adjusted for variables in the main model and additional variables that may be potential confounders and precision variables. These were paternal history of atopy, prenatal smoking, prenatal cat or dog ownership, delivery type, breastfeeding duration, and prenatal antibiotics use. Paternal history of atopy was included in only the extended model because its association with child AD is less well-established than the association between maternal history of atopy and child AD [28]. In secondary analyses, we presented all model estimates of the association between prenatal SLEs and our secondary outcome, ever AD. We assessed effect modification of the relationship between prenatal SLEs and primary outcomes by child sex, maternal history of atopy, and prenatal maternal resilient coping using interaction terms for prenatal SLE exposure and the potential effect modifier of interest in the separate main models. As a sensitivity analysis, we conducted deletion diagnostics to address potentially highly influential observations of reported prenatal SLEs. All analyses were conducted using RStudio version 3.6.1 and statistical significance was evaluated at an *alpha* = 0.05 level.

## 3. Results

The final sample size was 426 dyads after excluding 7 dyads due to incomplete responses on the PRAMS SLE survey. No dyads had missing outcome data. Among women in the overall sample, 56.3% had completed a college degree or more, 42.3% had a household income of USD 80,000 or more, 42.5% had a history of atopy, 80.0% were White, and the median (IQR) age at delivery was 31.0 (27.0–34.0) years (Table 1). Among children, 48.8% were male, 36.2% were born by cesarean section, and the median (IQR) child age at the follow-up visit was 5.5 (5.1–6.1) years.

Based on a median of 1 prenatal SLE experienced by women in our sample, we assessed differences in sociodemographic, health, and pregnancy-related characteristics between women reporting no SLEs (*N* = 158) and women reporting ≥1 prenatal SLEs (*N* = 268). Compared to women reporting no prenatal SLEs, women reporting ≥1 prenatal SLE more frequently had a high school degree or less, had a household income of less than USD 50,000, and were more racially and ethnically diverse (Table 1). Women experiencing ≥1 prenatal SLEs also more commonly had a history of atopy and were less likely to breastfeed their child for more than 6 months relative to women who experienced no SLEs.

The mean (standard deviation) of prenatal SLEs reported in the overall sample was 1.4 (1.6) (Table 2). The proportions of women reporting a total sum of 0, 1, and ≥2 prenatal SLEs were 37.1%, 26.3%, and 36.6%, respectively. The most common prenatal SLEs that women reported were moving to a new address (25.1%), having a sick family member in the hospital (18.5%), and arguing more than usual with their partner (17.4%). Women in the overall sample least frequently reported being homeless (1.2%), going to jail or partner going to jail (2.1%), and separating or divorcing from their partner (3.5%) (Table 2).

**Figure 1:**
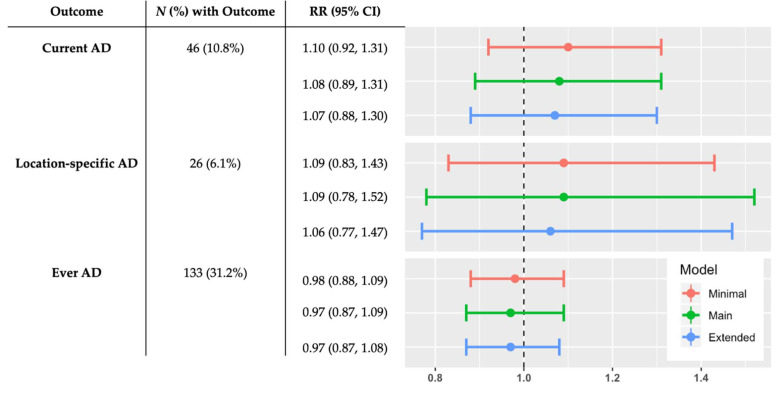
Sample frequencies of outcomes and adjusted associations between prenatal stressful life events and atopic dermatitis outcomes. Minimal model adjusts for child sex, child age, maternal education, household income, maternal race, maternal ethnicity, and maternal history of atopy. Main model adjusts for minimal model covariates and maternal age at delivery, maternal current stress, recruitment site, other children in home, and prenatal farm animal exposure. Extended model adjusts for main model covariates and paternal history of atopy, prenatal smoking, prenatal cat or dog ownership, delivery type, breastfeeding duration, and prenatal antibiotics. Abbreviations: AD, atopic dermatitis; CI, confidence interval; RR, risk ratio.

The prevalence of current AD, location-specific AD, and ever AD was 10.8%, 6.1%, and 31.2%, respectively (Figure 1). The Seattle and Yakima recruitment sites had similar current AD (10.8% vs. 10.5%, respectively) location-specific AD (7.0% vs. 5.0%, respectively), and ever AD prevalence (29.6% vs. 31.8%, respectively). Crude model estimates of the association between prenatal SLEs and all child AD outcomes were not statistically significant (data not shown). Based on main model estimates, we found that a 1-unit increase in reported prenatal SLEs was not significantly associated with current AD (RR: 1.08, 95% CI: 0.89, 1.31), location-specific AD (RR: 1.09, 95% CI: 0.78, 1.52), or ever AD (RR: 0.97, 95% CI: 0.87, 1.09) in children aged approximately 4–6 years (Figure 1).

In secondary analyses, we assessed whether there was effect modification by child sex, maternal history of atopy, or maternal prenatal resilient coping in the association between prenatal SLEs and current AD or location-specific AD. We found there was no evidence to suggest that the associations between prenatal SLEs and primary outcomes differed when stratified by child sex (current AD *p* for interaction = 0.96; location-specific AD *p* for interaction = 0.59), maternal history of atopy (current AD *p* for interaction = 0.98; location-specific AD *p* for interaction = 0.97), or maternal prenatal resilient coping (current AD *p* for interaction = 0.20; location-specific AD *p* for interaction = 0.54) (Table 3).

Finally, we ran a sensitivity analysis to assess whether potentially outlying prenatal SLE observations were driving the associations found in primary analyses. After deleting the highest reported observation of nine prenatal SLEs, we found RR estimates were lower than original estimates but not statistically significant for both current AD (RR: 1.00, 95% CI: 0.83, 1.20) and location-specific AD (RR: 0.92, 95% CI: 0.68, 1.23).

## 4. Discussion

In this prospective pregnancy cohort study, we found no associations between maternal experience of prenatal SLEs and children’s current AD, location-specific AD, and ever AD in children aged approximately 4–6 years. Results from our effect modification analyses suggest that contrary to our hypotheses, these relationships did not differ significantly by child sex, maternal history of atopy, and maternal report of resilient coping strategies during pregnancy.

The null associations we found between prenatal stress and child AD align with two previous longitudinal studies that followed children from a young age until middle childhood or early adolescence and similarly reported null associations at ages 4 to 6 years [7,16]. However, null findings form the minority of reported associations as the existing literature has generally shown a positive relationship between prenatal stress and child AD. Evidence from the majority of prior epidemiologic studies suggests a variety of prenatal stress and psychological functioning measures are associated with child AD, including SLEs [6,7], job strain [11], anxiety and depression [9,12,13,14], and general psychological distress [29]. Findings from the current study are inconsistent with the two previous prenatal SLE analyses, each of which found positive associations between prenatal SLEs and child AD in children aged 8.5 years, on average [6], and 14 years [7].

One reason we did not detect an association between prenatal SLEs and child AD may be that we sampled children aged approximately 4–6 years, and phenotypic differences in AD in very early life versus early and later childhood may have differing relationships with prenatal stress-related programming. There is evidence that AD that persists beyond infancy and toddlerhood is associated with more severe disease [17]. One longitudinal study reported an association in young children up to 2 years but not in children aged 4–6 years, suggesting the influence of prenatal stress may be most important for early life AD [16]. Other factors besides prenatal stress may be key in driving associations either at later ages or in individuals with more severe disease [16,30]. For instance, the potential influence of postnatal maternal stress on child AD is supported by findings from one prior study that reported 86% greater odds (95% CI: 1.09, 3.19) of AD in children aged 4 years who experienced parental divorce or separation in the first 2 years of life [31]. However, a different study found no association between prenatal SLEs and AD in children at 6 years of age but reported a positive relationship in the same children at age 14 [7]. This study noted that a substantial number of AD cases at age 6 years were transient and had resolved by age 14 years, suggesting that prenatal stress may have a greater effect on AD that persists through middle childhood and into adolescence but not on AD that resolves before then. These differences in associations across studies may reflect phenotypic differences in childhood AD that are partially captured by severity, age at onset, and resolution, and indicate the need for future longitudinal research [30].

A major strength of this study was that it was a multicenter prospective cohort and the first analysis to assess the association between prenatal stress and child AD in the U.S., which is important given potential differences in stress exposure for pregnant women from countries offering paid maternity leave and employment protection services. We also were able to adjust for a suite of important potentially confounding variables, control of which has been more limited in prior studies. Sociodemographic factors including maternal race, ethnicity, education, and income were not accounted for in several studies reporting positive associations [11,13,14], and only two earlier analyses adjusted for postnatal stress [7,9]. Although inclusion of postnatal stress in our adjustment models did not change risk estimates, it is possible that unmeasured confounding by these potentially influential variables may have biased estimates reported in previous studies. Other strengths of the present analysis included examining potential effect modifiers that had yet to be explored, administering the validated PRAMS SLE survey to capture our exposure, and using the validated ISAAC questionnaire for our outcome assessments. The prevalence of current AD in our sample (10.8%) corresponded to previously reported estimates in Washington state (10.7%) and the U.S. (10.6%) [32].

Our study is subject to limitations. First, our sample had a lower mean of reported prenatal SLEs experienced (1.4 SLEs) compared to Washington state (1.7 SLEs) and U.S. (1.8 SLEs) mean estimates [33]. Additionally, the frequencies of affirmative responses to individual prenatal SLE items in the CDC PRAMS questionnaire were generally lower in our sample relative to other studies [23,34,35,36]. Compared to the current analysis, studies with higher frequencies of reported prenatal SLEs sampled individuals who less frequently identified as White and had a lower completed level of education. The opportunity to identify a statistically significant association may have been reduced given the lack of socioeconomic diversity and, correspondingly, less broad distribution of reported prenatal SLEs in our study population. The large proportion of women in our sample who were White and highly educated also limits the generalizability of our findings.

Additionally, while our power calculations supported an opportunity to see effect sizes reported in other studies, our sample size and minimal maternal exposure to SLEs did limit our power to detect more modest associations in main and secondary analyses, test for other effect modifiers of interest, and conduct sensitivity analyses of associations differing by prenatal SLE type.

Finally, like most previous studies assessing the relationship between prenatal stress and child AD, we relied on parental report for both prenatal SLE assessment and symptoms to define child AD outcomes. Although the CDC PRAMS survey ascertains prenatal SLE exposure information retrospectively, this instrument has been shown to be accurate over time and robust to recall bias [37]. Parental report of child AD may be subject to a greater degree of bias, however. In a study conducted by Braig et al., prenatal anxiety was associated with a 40% greater risk of child AD when AD was defined according to parental report of symptoms (RR: 1.4, 95% CI: 1.0, 2.0) [14]. However, when the authors changed the outcome to meet stricter criteria of concurrent parental- and pediatrician-diagnosed AD, they found the relationship was no longer significant (RR: 1.1, 95% CI: 0.7, 1.9). It is possible that, in the Braig et al. study and others defining AD according to parental report, a substantial number of women who experienced prenatal stress misclassify their children as having AD. This differential misclassification with respect to the outcome could lead to spuriously high reported associations compared to true associations. It is also possible that child age and associated phenotypic differences may play a role in the degree of misclassification present in report of child AD. Braig et al. followed children up to 2 years of age, a population that may exhibit AD that differs phenotypically from that of preschoolers and young school-age children. Future studies are needed to compare associations between prenatal stress and child AD, with AD defined according both to parental report and more objective diagnostic criteria.

AD remains a very common problem in childhood and prevalence has steadily increased over time in the U.S. [38]. Results from our analysis contribute to both a relatively limited literature on prenatal stress and child AD as well as to a wider scientific community interest in the role of maternal stress in development of all allergic diseases. Future studies that include large, well-characterized, and ethnically diverse samples, describe disease along the childhood life course, and assess disease with both symptom reports and reports of diagnosis will be most informative for enhanced understanding of the role of prenatal stress in child AD.

## 5. Conclusions

This was the first study conducted in the U.S. that evaluated the relationship between prenatal maternal stress and AD in children and the first to explore potential effect modification by maternal resilience. After adjusting for potential confounders, results suggested that there were no statistically significant associations between prenatal SLEs and current, location-specific, or ever AD in children aged approximately 4–6 years. Further research in this understudied field should examine this relationship in large longitudinal cohorts with diverse child age ranges and carefully consider assessment of AD outcomes.

## Figures and Tables

**Table 1:** Sociodemographic characteristics of dyads enrolled in the Global Alliance to Prevent Prematurity and Stillbirth (GAPPS) Repository with subsequent enrollment in the Environmental influences on Child Health Outcomes (ECHO) PATHWAYS approximately aged 4 to 6-year visit (*N* = 426).

Characteristic	Overall *N* = 426	0 Prenatal SLEs ^1^ *N* = 158	≥1 Prenatal SLEs *N* = 268
	Median (IQR)	Median (IQR)	Median (IQR)
Child age (years)	5.5 (5.1–6.1)	5.5 (5.1–6.0)	5.5 (5.2–6.1)
Maternal age (years)	31.0 (27.0–34.0)	32.0 (28.0–35.0)	30.5 (27.0–34.0)
Missing, *N* (%)	4 (0.9%)	2 (1.3%)	2 (0.7%)
Maternal current stress (score on PSS) ^2^	9.0 (7.0–11.0)	8.0 (6.3–10.0)	9.0 (8.0–11.0)
Child sex	N (%)	N (%)	N (%)
Male	208 (48.8)	74 (46.8)	134 (50.0)
Female	218 (51.2)	84 (53.2)	134 (50.0)
Maternal education			
<High school degree	15 (3.5)	2 (1.3)	13 (4.9)
High school graduate or GED	39 (9.2)	9 (5.7)	30 (11.2)
Some college or technical/ vocational school graduate	119 (27.9)	39 (24.7)	80 (29.9)
≥College degree	240 (56.3)	104 (65.8)	136 (50.7)
Missing	13 (3.1)	4 (2.5)	9 (3.3)
Prenatal household income			
<USD 50,000	129 (30.3)	34 (21.5)	95 (35.6)
USD 50,000–79,999	84 (19.7)	40 (25.3)	44 (16.4)
≥USD 80,000	180 (42.3)	76 (48.1)	104 (38.8)
Missing	33 (7.7)	8 (5.1)	25 (9.3)
Maternal history of atopy			
No	245 (57.5)	95 (60.1)	150 (56.0)
Yes	181 (42.5)	63 (39.9)	118 (44.0)
Paternal history of atopy			
No	291 (68.3)	114 (72.2)	177 (66.0)
Yes	135 (31.7)	44 (27.8)	91 (34.0)
Maternal report of race			
White	341 (80.0)	136 (86.1)	205 (76.4)
Black or African American	8 (1.9)	0 (0)	8 (3.0)
Asian	12 (2.8)	4 (2.5)	8 (3.0)
American Indian/Alaskan Native	4 (0.9)	0 (0)	4 (1.5)
Multiple Race	33 (7.8)	9 (5.7)	24 (9.0)
Other	12 (2.8)	4 (2.5)	8 (3.0)
Missing	16 (3.8)	5 (3.2)	11 (4.1)
Maternal report of ethnicity			
Not Hispanic/Latino	363 (85.2)	142 (89.9)	221 (82.5)
Hispanic/Latino	60 (14.1)	16 (10.1)	44 (16.4)
Missing	3 (0.7)	0 (0)	3 (1.1)
Other children living in home			
No	52 (12.2)	19 (12.0)	33 (12.3)
Yes	374 (87.8)	139 (88.0)	235 (87.7)
Recruitment site ^3^			
Seattle	206 (48.4)	78 (49.4)	128 (47.8)
Yakima	220 (51.6)	80 (50.6)	140 (52.2)
Prenatal farm animal exposure			
No	400 (93.9)	149 (94.3)	251 (93.7)
Yes	25 (5.9)	9 (5.7)	16 (6.0)
Missing	1 (0.2)	0 (0)	1 (0.3)
Prenatal cat or dog ownership			
No	183 (43.0)	67 (42.4)	116 (43.3)
Yes	243 (57.0)	91 (57.6)	152 (56.7)
Prenatal smoking			
No	401 (94.1)	150 (94.9)	251 (93.7)
Yes	12 (2.8)	3 (1.9)	9 (3.3)
Missing	13 (3.1)	5 (3.2)	8 (3.0)
Delivery type			
Vaginal	272 (63.8)	102 (64.6)	170 (63.4)
Cesarean	154 (36.2)	56 (35.4)	98 (36.6)
Breastfeeding duration			
Did not breastfeed	25 (5.9)	7 (4.4)	18 (6.7)
>0 to <6 months	133 (31.2)	46 (29.1)	87 (32.5)
>6 months	265 (62.2)	104 (65.8)	161 (60.1)
Missing	3 (0.7)	1 (0.7)	2 (0.7)
Prenatal antibiotics			
No	419 (98.4)	154 (97.5)	265 (98.9)
Yes	7 (1.6)	4 (2.5)	3 (1.1)
Maternal resilient coping ^4^			
Low resilient coping	129 (30.3)	44 (27.8)	85 (31.7)
Medium resilient coping	145 (34.0)	57 (36.1)	88 (32.8)
High resilient coping	133 (31.2)	52 (32.9)	81 (30.3)
Missing	19 (4.5)	5 (3.2)	14 (5.2)

Abbreviations: PSS, Cohen’s Perceived Stress Scale; SD, standard deviation; SLE, stressful life event. ^1^ Prenatal SLE groups categorized in reference to median number of SLEs experienced (0, below median; ≥1, at or above median). ^2^ Participants who answered all four items on the PSS could receive a possible score between 4 and 20, where higher scores indicated greater stress. ^3^ Seattle recruitment sites: University of Washington Medical Center and Swedish Medical Center; Yakima recruitment site: Yakima Valley Memorial Hospital. ^4^ Maternal resilient coping defined according to score on four-item Brief Resilient Coping Scale (4–13, low resilient coping; 14–16, medium resilient coping; 17–20, high resilient coping).

**Table 2:** Frequencies of maternal report of prenatal stressful life event types by questionnaire item and total sum.

Reported Total Sum of Prenatal SLEs, Mean (SD)	1.4 (1.6)
Reported Total Sum of prenatal SLEs, range	0–9
Specific Prenatal SLE	*N* (%)
Moved addresses	107 (25.1%)
Sick family member in hospital	79 (18.5%)
More arguments than usual with partner	74 (17.4%)
Problems paying bills	59 (13.8%)
Someone close had drinking or drug problem	57 (13.4%)
Someone close died	48 (11.3%)
Cut in work hours or pay	43 (10.1%)
Partner lost job	30 (7.0%)
Partner did not want pregnancy	25 (5.9%)
Lost job	22 (5.2%)
Partner deployed	18 (4.2%)
Separation or divorce	15 (3.5%)
Partner or self-jailed	9 (2.1%)
Homelessness	5 (1.2%)

Abbreviations: SD, standard deviation; SLE, stressful life event.

**Table 3:** Adjusted associations between prenatal stressful life events and child atopic dermatitis outcomes across strata of child sex, maternal history of atopy, and prenatal maternal resilient coping category.

Characteristic	Current AD ^1,2^ RR (95% CI)	Location-Specific AD ^1,2^ RR (95% CI)
**Child sex**		
Male	1.07 (0.82, 1.41)	1.14 (0.77, 1.72)
Female	1.08 (0.80, 1.47)	0.95 (0.54, 1.69)
p for interaction	0.96	0.59
**Maternal history of atopy**		
Yes	1.08 (0.84, 1.38)	1.09 (0.77, 1.54)
No	1.07 (0.84, 1.37)	1.08 (0.71, 1.64)
p for interaction	0.98	0.97
**Prenatal maternal resilient coping ^3^**		
Low resilient coping	0.89 (0.69, 1.14)	1.00 (0.68, 1.46)
Medium resilient coping	1.25 (0.79, 1.97)	0.87 (0.41, 1.87)
High resilient coping	1.28 (0.94, 1.74)	1.29 (0.87, 1.92)
p for interaction	0.20	0.54

Abbreviations: AD, atopic dermatitis; CI, confidence interval; RR, risk ratio. ^1^ Estimates are from main models which adjust for child sex, child age, maternal education, household income, maternal race, maternal ethnicity, maternal history of atopy, maternal age at delivery, maternal current stress, recruitment site, other children in home, and prenatal farm animal exposure. ^2^ All RRs estimate the association between prenatal SLEs and child AD corresponding to a 1-unit increase in reported prenatal SLE sum. ^3^ Maternal resilient coping defined according to score on 4-item Brief Resilient Coping Scale (4–13, low resilient coping; 14–16, medium resilient coping; 17–20, high resilient coping).

## Data Availability

The data utilized for this study are not publicly available but de-identified data may be available upon request, subject to approval by the internal review board and under a formal data use agreement. Contact the corresponding author for more information.
